# Impact of planned dose reporting methods on Gamma pass rates for IROC lung and liver motion phantoms treated with pencil beam scanning protons

**DOI:** 10.1186/s13014-019-1316-y

**Published:** 2019-06-17

**Authors:** Yixiu Kang, Jiajian Shen, Wei Liu, Paige A. Taylor, Hunter S. Mehrens, Xiaoning Ding, Yanle Hu, Erik Tryggestad, Sameer R. Keole, Steven E. Schild, William W. Wong, Mirek Fatyga, Martin Bues

**Affiliations:** 1 Department of Radiation Oncology, Mayo Clinic, Phoenix, AZ 85054 USA; 2 Seattle Cancer Care Alliance Proton Therapy Center, 1570 N 115th St, Seattle, WA 98133 USA; 3 The Imaging and Radiation Oncology Core Houston Quality Assurance Center, The University of Texas MD Anderson Cancer Center, Unit 607, 1515 Holcombe Blvd, Houston, TX 77030 USA; 4 Department of Radiation Oncology, Mayo Clinic, Rochester, MN 55905 USA

**Keywords:** Proton therapy, Pencil beam scanning proton, Motion phantom, Lung phantom, Liver phantom

## Abstract

**Purpose:**

The purpose of this study is to evaluate the impact of two methods of reporting planned dose distributions on the Gamma analysis pass rates for comparison with measured 2D film dose and simulated delivered 3D dose for proton pencil beam scanning treatment of the Imaging and Radiation Oncology Core (IROC) proton lung and liver mobile phantoms.

**Methods and materials:**

Four-dimensional (4D) computed-tomography (CT) image sets were acquired for IROC proton lung and liver mobile phantoms, which include dosimetry inserts that contains targets, thermoluminescent dosimeters and EBT2 films for plan dose verification. 4DCT measured fixed motion magnitudes were 1.3 and 1.0 cm for the lung and liver phantoms, respectively. To study the effects of motion magnitude on the Gamma analysis pass rate, three motion magnitudes for each phantom were simulated by creating virtual 4DCT image sets with motion magnitudes scaled from the scanned phantom motion by 50, 100, and 200%. The internal target volumes were contoured on the maximum intensity projection CTs of the 4DCTs for the lung phantom and on the minimum intensity projection CTs of the 4DCTs for the liver phantom. Treatment plans were optimized on the average intensity projection (AVE) CTs of the 4DCTs using the RayStation treatment planning system. Plan doses were calculated on the AVE CTs, which was defined as the planned AVE dose (method one). Plan doses were also calculated on all 10 phase CTs of the 4DCTs and were registered using target alignment to and equal-weight-summed on the 50% phase (T50) CT, which was defined as the planned 4D dose (method two). The planned AVE doses and 4D doses for phantom treatment were reported to IROC, and the 2D-2D Gamma analysis pass rates for measured film dose relative to the planned AVE and 4D doses were compared. To evaluate motion interplay effects, simulated delivered doses were calculated for each plan by sorting spots into corresponding respiratory phases using spot delivery time recorded in the log files by the beam delivery system to calculate each phase dose and accumulate dose to the T50 CTs. Ten random beam starting phases were used for each beam to obtain the range of the simulated delivered dose distributions. 3D-3D Gamma analyses were performed to compare the planned 4D/AVE doses with simulated delivered doses.

**Results:**

The planned 4D dose matched better with the measured 2D film dose and simulated delivered 3D dose than the planned AVE dose. Using planned 4D dose as institution reported planned dose to IROC improved IROC film dose 2D-2D Gamma analysis pass rate from 92 to 96% on average for three films for the lung phantom (7% 5 mm), and from 92 to 94% in the sagittal plane for the liver phantom (7% 4 mm), respectively, compared with using the planned AVE dose. The 3D-3D Gamma analysis (3% 3 mm) pass rate showed that the simulated delivered doses for lung and liver phantoms using 10 random beam starting phases for each delivered beam matched the planned 4D dose significantly better than the planned AVE dose for phantom motions larger than 1 cm (*p ≤* 0.04).

**Conclusions:**

It is recommended to use the planned 4D dose as the institution reported planned dose to IROC to compare with the measured film dose for proton mobile phantoms to improve film Gamma analysis pass rate in the IROC credentialing process.

## Introduction

Credentialing by the Imaging and Radiation Oncology Core (IROC) Houston Quality Assurance Center is required for proton centers to participate in National Cancer Institute funded clinical trials. The IROC moving proton phantoms were designed to simulate patient respiratory motion and tissue heterogeneity. Proton centers are asked to plan and treat the IROC phantoms as they would treat protocol patients. IROC recently reported that many proton centers failed their dosimetry tests for phantoms with motion and heterogeneity [1, 2]. Many factors, such as the target motion interplay effect, dose calculation accuracy of the treatment planning system (TPS), and how to use four-dimensional (4D) computed-tomography (CT) to calculate and report the planned dose, can all contribute to credentialing failure when mobile targets are treated using pencil beam scanning (PBS) proton beams.

Several strategies, such as volumetric and layer repainting [3–9], gating [10, 11], robust optimization [12–15], and optimizing spot delivery sequence [16], have been proposed to mitigate target motion interplay effect. Li *et al* showed that dynamically accumulated dose with interplay effect considered will converge to the 4D dose after multiple deliveries for irradiation of moving tumors regardless of treatment modality and delivery properties [17]. Therefore, delivered dose would converge to 4D dose if target motion interplay effects can be mitigated adequately. Use of Monte Carlo based dose calculation engines is also recommended for addressing the heterogeneity of the IROC lung phantom for PBS [2]. Proton treatment planning and dose volume histogram (DVH) parameter evaluation using the average intensity (AVE) CT of the 4DCT with target density override is an effective and practical method to avoid a more complex and time consuming 4D dose calculation [18]. However, the impact of using AVE dose versus 4D dose when comparing planned dose with measured dose distributions has not been reported. Simulated delivered dose which includes spot delivery time information in dose calculation can also be used as a dose reporting method for PBS. However, calculating simulated delivered dose requires beam starting phase and the realistic motion pattern, which may vary each delivery and cannot be obtained at the planning stage. The delivered dose estimation at the planning stage can be one of the dose distributions in a range of dose distributions with the average converging to 4D dose [17]. Therefore, a single simulated delivered dose may not be the best representation of the delivered dose and the average of the simulated delivered doses using multiple beam starting phases is similar to 4D dose.

The IROC mobile proton phantoms were designed to simulate patient respiratory motion and tissue equivalent materials for proton stopping power. The proton centers using IROC credentialing process are asked to plan and treat the phantoms just as they would treat protocol patients. Therefore, failed phantom treatment may relate to a possible poor outcome for patients in clinical trials. In this study, we quantitatively evaluated the impact of using the AVE (method one) and 4D dose (method two) as planned dose to compare with measured film dose and simulated delivered dose on the Gamma analysis pass rates of the IROC proton lung and liver mobile phantoms. Our work is intended to help effectively improve the accuracy of the dosimetry comparison for IROC mobile proton phantoms, and help proton centers pass the IROC credentialing process for PBS treatments of moving phantoms.

## Methods and materials

### Phantom description and 4DCT

**Fig. 1:**
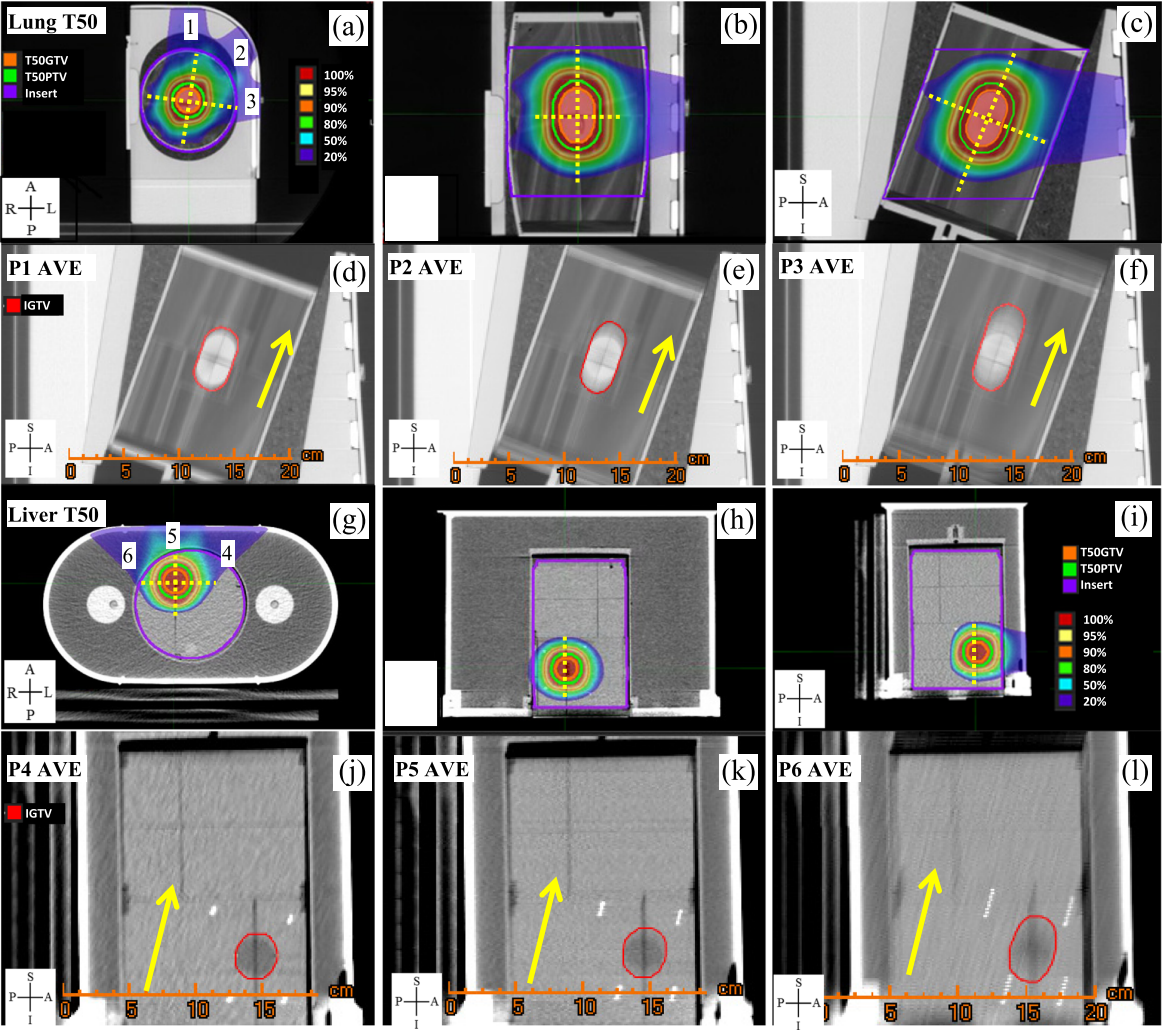
T50 CT of the 4DCT for the lung/liver phantom in the (**a**)/(**g**) axial, (**b**)/(**h**) coronal, and (**c**)/(**i**) sagittal view, respectively. AVE CTs in the sagittal view for the lung/liver phantom P1/P4, P2/P5, and P3/P6 with motion magnitude of (**d**)/(**j**) 0.7/0.5 cm, (**e**)/(**k**) 1.3/1.0 cm, and (**f**)/(**l**) 2.6/2.0 cm, respectively. The numbers 1, 2, and 3 in (**a**), and 4, 5, and 6 in (**g**) indicated proton treatment beam directions. The yellow arrows and dotted lines in the figures indicated the motion direction of the dosimetry inserts and the film locations (three films in the lung phantom and two films in the liver phantom) in the dosimetry inserts, respectively. Abbreviations: T50, 50% phase; GTV, gross tumor volume; PTV planning target volume; IGTV, internal gross tumor volume; AVE, average intensity

For this study, we used the proton lung (Fig. 1a-f) and liver (Fig. 1g-l) mobile anthropomorphic phantoms provided by IROC, Houston for proton center credentialing to participate in clinical trials [1]. The phantoms include dosimetry inserts that contains targets, thermoluminescent dosimeters (TLD) and EBT2 films. The TLDs and films were used for absolute dose and 2D dose comparison, respectively [1]. The pre-programmed fixed motion magnitudes were 2.0 and 1.0 cm and the recorded respiratory cycles were 4.3 and 5.0 s, for the lung and liver phantoms, respectively. Ten phase equal-time-spaced 4DCT scans with slice thickness of 1.25 mm were acquired for both phantoms using a GE Optima 580 CT scanner with Anzai belt used to record respiratory trace for 4DCT image sorting (GE Healthcare, Waukesha, WI). Because the motion was suppressed by the Anzai belt, the lung phantom target motion measured on the scanned 4DCT was 1.3 cm, which was less than the ruler measured motion magnitude of 2 cm for phantom treatment when the Anzai belt was not used. As the center practice at the Seattle Proton Therapy Center, Anzai belt was used only for 4DCT to sort images but not for patient treatment. To follow the clinic practice as recommend by IROC, the phantom was treated without Anzai belt. The displacement vectors between the T50 phase and each of the other nine phases of the scanned 4DCT were measured by registering images using the dosimetry insert. The image registration accuracy was within 1 mm. To study the effects of the motion magnitude on Gamma analysis pass rates, six virtual phantom 4DCT image sets (Fig. 1) were constructed for lung (P1, P2, and P3) and liver (P4, P5, and P6) phantoms by scaling the CT-scanned phantom motion magnitude of each 4DCT phase relative to the 50% phase (T50) CT of the 4DCT by 50, 100, and 200% to simulate small (P1/P4), medium (P2/P5), and large (P3/P6) magnitude of target motion. Virtual phantom 4DCT images were obtained using an in-house WinPython 3.4.3.1 tool by shifting the mobile components of the phantoms from the T50 CT to the corresponding phase CT voxel by voxel. The T50 CT was used to generate all phase CT image sets instead of scanned phase CT to reduce the motion artifacts because the T50 CT showed minimum motion artifacts among all phase CTs. Reducing the motion artifacts of each phase CT improves dose calculation accuracy on each phase CT. Compared to the scanned 4DCT, the 100% scaled CT had the same motion magnitude as the scanned CT data set for each phase CT but without the dosimetry insert/target motion blurring. For each of the 6 4DCT image sets, the maximum intensity projection (MIP), minimum intensity projection (min-IP), and average intensity projection (AVE) CT image sets were constructed using MIM 6.4.3 (MIM Software Inc., Cleveland, OH). The T50 CT was selected as the reference phase for 4D dose accumulation because the T50 CT is the most stable phase and showed minimum motion artifacts among all 10 phases of the 4DCT.

### Target definition and treatment planning

The gross tumor volume (GTV) was contoured on the T50 CT as T50GTV. The internal GTV (IGTV) was contoured on the MIP CT for the lung phantom and on the min-IP CT for the liver phantom. The T50 CT planning target volume (T50PTV) was defined as the T50GTV plus a 5 mm margin, and the PTV was defined as the IGTV plus a 5 mm margin to take into account the inter-fractional motion, intra-fractional motion, and setup uncertainty. The IGTV and PTV were used for plan optimization and target dose evaluation on AVE CT, and the T50GTV and T50PTV were used on T50 CT for 4D target dose evaluation. Only 1 (PTV 2) of the 2 IROC liver phantom targets was selected for this study because both targets had the same motion magnitude and similar planned beam characteristics. The IGTV density override method [18] was used for the lung phantom plan. No density override was used for the liver phantom plan because of the small density variation between the target and surrounding tissues. Per IROC instruction, the planning goal was to have 6 Gy(relative biological effectiveness [RBE]) prescription dose to at least 95% of the PTV and a minimum dose of 5.4 Gy(RBE) to at least 99% of the PTV. For each phantom motion, a PBS plan with three coplanar beams was optimized on the AVE CT using robust optimization in RayStation 4.5 TPS (RaySearch Laboratories, Stockholm, Sweden). The dose calculation accuracy of the TPS was reported in the center TPS commission report [19]. The target sizes, gantry angles (G), table angles (T), beam energies (E), number of layers, and monitor units (MU) of each plan are listed in Table 1. Each beam delivered 2 Gy(RBE) uniform target dose. Each plan dose was calculated on the AVE CT and defined as the planned AVE dose. Each plan dose was also calculated on each phase CT of the virtual 4DCT and was rigidly registered using insert/target alignment to and equal-weight-summed on the T50 CT, which was defined as the planned 4D dose.

**Table 1:** Lung (P1, P2, and P3) and liver (P4, P5, and P6) phantom plan target and beam parameters

		Lung Phantom (T50GTV/T50PTV: 31.1/66.0 cm^3^)			Liver Phantom (T50GTV/T50PTV: 15.4/37.9 cm^3^)			
		P1	P2	P3		P4	P5	P6
Motion	(cm)	0.7	1.3	2.6		0.5	1.0	2.0
IGTV	(cm^3^)	37.4	38.8	51.9		18.9	23.0	30.1
PTV	(cm^3^)	77.1	80.5	102.9		44.0	51.4	63.9
Beam 1	MU	620.1	659.0	766.9	Beam 4	588.7	635.0	772.3
(G0 T0)	Layer	16	17	18	(G50 T0)	29	29	29
	E (MeV)	147.1–109.3	147.1–107.0	147.1–104.7		162.1–99.8	162.1–99.8	162.1–99.8
Beam 2	MU	625.8	667.2	777.1	Beam 5	593.9	634.9	760.7
(G45 T0)	Layer	15	14	15	(G0 T0)	17	21	21
	E (MeV)	144.4–109.3	144.4–111.5	144.4–109.3		150.1–113.7	150.1–105.6	150.1–105.6
Beam 3	MU	633.8	675.5	783.4	Beam 6	591.7	635.3	767.8
(G90 T0)	Layer	15	13	15	(G310 T0)	26	26	26
	E (MeV)	139.0–104.7	136.3–107.0	139.0–104.7		155.1–99.8	155.1–99.8	155.1–99.8

*Abbreviations*: *T50* 50% phase, *GTV* gross tumor volume, *IGTV* internal gross tumor volume, *PTV* planning target volume, *E* energy, *MU* monitor unit, *G* gantry, *T* table

### PBS beam delivery and IROC film dose analysis

The IBA Proteus 230 PBS gantry with a universal nozzle was used to deliver the PBS beams. The range, energy, and in-air spot size at the iso-center of the system were 8.0 to 32.0 cm, 98.5 to 228.5 MeV, and 7.3 to 3.3 mm (1 sigma), respectively. Two range shifters, 4.0 and 7.5 g/cm^2^, were also available to treat shallow targets. The PBS beams were delivered layer-by-layer from the highest to the lowest energy and the spots were delivered row-by-row from bottom to top in each layer and from left to right in each row in beams-eye-view using step and shoot delivery. The PBS spot delivery parameters (including spot position, charge collected by MU chambers, etc.) were recorded every 250 microseconds during beam delivery by the beam delivery system in a log file for each beam delivery.

For center credentialing, the lung phantom (P2) was treated using three planned 1 Gy(RBE) beams, each delivered twice, and the liver phantom (P5) was treated using three planned 2 Gy(RBE) beams, each delivered once. Phantom (P2 and P5) treatment setup used orthogonal kV X-ray images to align the non-moving parts of the phantoms to the digitally reconstructed radiography of the phantoms from the planning CTs, analogous to the 4DCT image registration for treatment planning. Quality assurance (QA) measurement was performed for each beam by applying the plan to a static solid water phantom and compare selected planar dose calculated by the TPS with the dose measured by an ion chamber array Matrixx PT (IBA Dosimetry) at a depth near the center of the water equivalent depth of the center of the target for each beam prior to the phantom treatments. To collect PBS spot delivery time data to calculate simulated delivered dose, the planned beams for P1, P3, P4, and P6 were also delivered to a solid water phantom.

Both planned AVE and 4D dose distributions for the lung and liver phantoms were sent to IROC, which were compared with the film doses for 2-dimensional (2D)-2D Gamma analysis pass rates. The planed doses were registered to the measured film doses using the center of the target based on the pin prick positions in the phantom. The combined uncertainty in the TLD and film dose measurements is 2.6 to 3.6%, and the spatial precision of the film and densitometer system is 1 mm [1, 2]. The Gamma criteria applied are the IROC standard for lung (7% 5 mm) and liver (7% 4 mm), and for research comparison, a stricter criteria of 3% 3 mm for both phantoms.

### Simulated delivered dose and 3-dimensional (3D)-3D gamma analysis

Since film measurements only provide 2D dose, the Gamma analysis pass rates were limited to fixed 2D planes. In order to compare the impact of the dose reporting methods on Gamma pass rates in 3D, delivered dose included spot delivery time information was simulated. The recorded spot delivery time data collected during beam delivery in the log files by the beam delivery system were used to sort PBS spots into the corresponding 4DCT phases using an in-house Python 3.4.3.1 tool and the doses were calculated on each corresponding 4DCT phase in RayStation TPS. The doses calculated on all 4DCT phases were rigidly registered using target alignment to and accumulated on the T50 CT, which was defined as simulated delivered dose. Ten random beam starting phases were used for each beam to estimate the range of dose distributions due to motion interplay effect. 3D-3D Gamma analyses were performed to compare the simulated delivered doses with planned 4D and AVE doses. The dose registration for Gamma analysis used the center of the target alignment to be consistent with IROC film dose Gamma analysis.

### Dose evaluation and statistical analysis

In this study, MIM 6.4.3–6.7.6 was used for dose summation and dosimetry parameter analyses. 3D-3D Gamma analysis [20] comparing the simulated delivered dose with the planned dose used in-house tools. 2D-2D Gamma analysis comparing film dose with planned dose performed by IROC used IROC in-house tools. 2D-2D Gamma analysis comparing Matrixx PT measured dose with planned dose for beam QA used OmniPro I’mRT software (IBA Dosimetry). The Gamma analysis used planned dose as reference dose and the threshold dose was 10% of 6 Gy(RBE) for 3D-3D Gamma and IROC film analysis, and 10% of the max planar dose for Matrixx PT beam QA. The paired Student *t* test was used to compare metrics between paired samples using MATLAB statistics toolbox (R2016a, The MathWorks, Inc., Natick, MA). A *p* value of less than 0.05 was considered statistically significant.

## Results

### Comparison of planned 4D and AVE doses

**Fig. 2:**
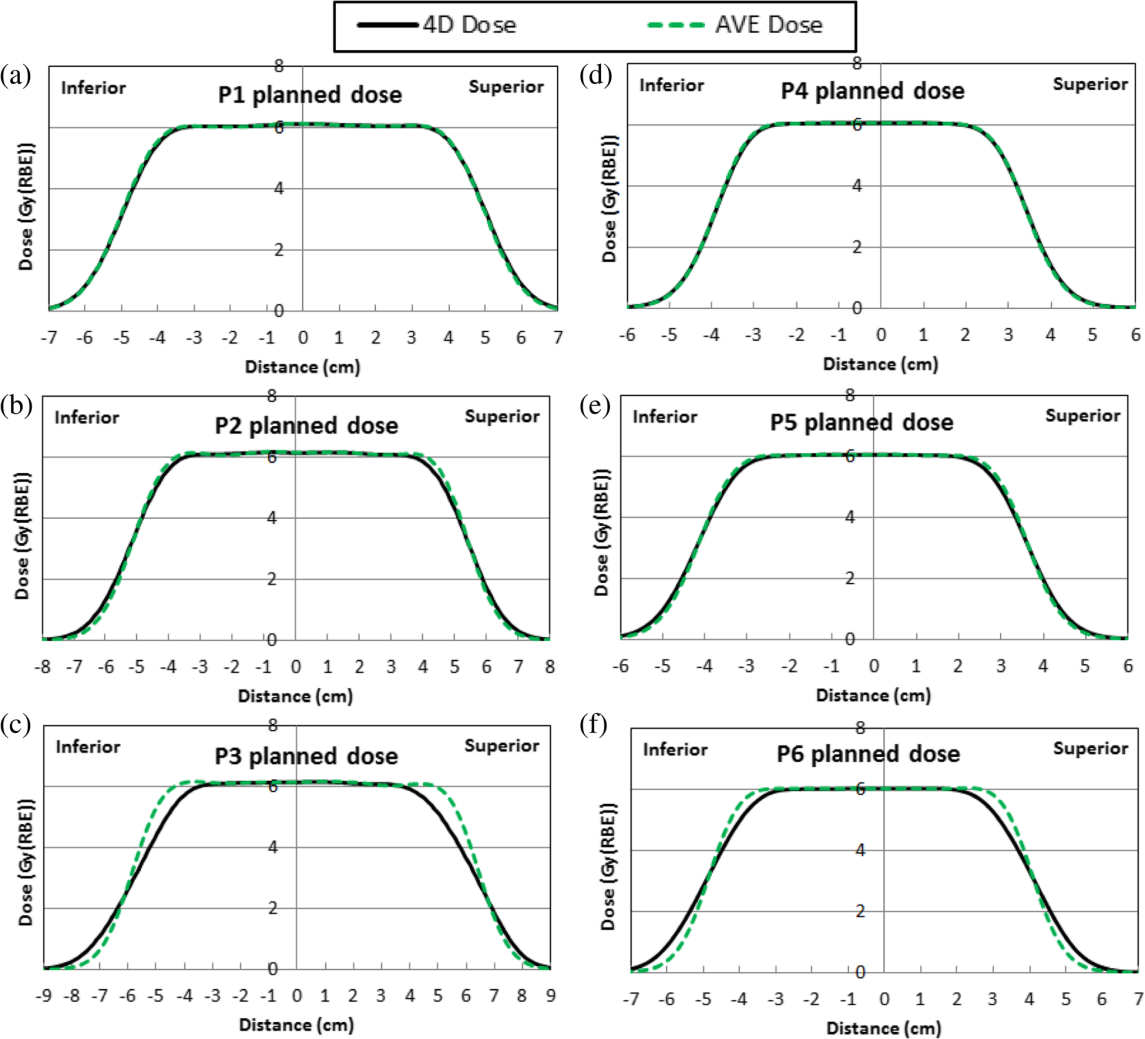
Planned 4D and AVE dose profiles in the center of the target in the target motion direction for the lung/liver phantom P1/P4, P2/P5, and P3/P6 with the motion magnitudes of (**a**)/(**d**) 0.7/0.5 cm, (**b**)/(**e**) 1.3/1.0 cm, and (**c**)/(**f**) 2.6/2.0 cm, respectively. 4D and AVE dose are aligned using the center of the target. Abbreviations: 4D, 4-dimensional; AVE, average intensity

Figure 2 shows the planned 4D and AVE lung and liver phantom dose profiles in the center of the target in the target motion direction. The AVE and 4D dose was aligned with each other using center of the targets, analogous to IROC film analysis when registering film dose to planned dose distributions. The planned 4D dose had a wider penumbra in the target motion direction than the planned AVE dose, and the difference ranged from 0.3 mm to 7.3 mm depending on the magnitudes of the target motion. Table 2 lists the average of the inferior and superior 20 to 80% (relative to the prescription dose) penumbras of the planned 4D/AVE dose profiles in the target motion direction and the difference of the penumbras between the 4D and AVE doses for P1, P2, P3, P4, P5, and P6, respectively. The difference of the penumbras in the target motion direction between the 4D and AVE doses increased as the target motion magnitude increased.

**Table 2:** The average of the inferior and superior 20 to 80% (relative to prescription dose) penumbra of the planned 4D/AVE dose profiles and the difference between 4D and AVE doses for lung (P1, P2, and P3) and liver (P4, P5, and P6) phantoms

		P1	P2	P3	P4	P5	P6
4D	(mm)	13.1	14.2	19.9	11.5	12.9	16.9
AVE	(mm)	12.6	12.2	12.6	11.2	11.4	11.5
Difference	(mm)	0.5	2.0	7.3	0.3	1.5	5.4

*Abbreviations*: *4D* 4-dimensional, *AVE* average intensity

### IROC phantom treatment QA and delivered film dose vs planned 4D and AVE doses

The pre-treatment beam QA for the lung phantom (P2) and liver phantom (P5) showed delivered dose measured by Matrixx PT agreed well with the planned dose for all beams in the measured planes. The 3% 3 mm 2D-2D Gamma analysis pass rate comparing the TPS calculated doses with the Matrixx PT measured doses for beams 1, 2, 3, 4, 5, and 6 were 99.3, 99.9, 99.5, 100.0, 100.0, and 99.0%, respectively.

**Fig. 3:**
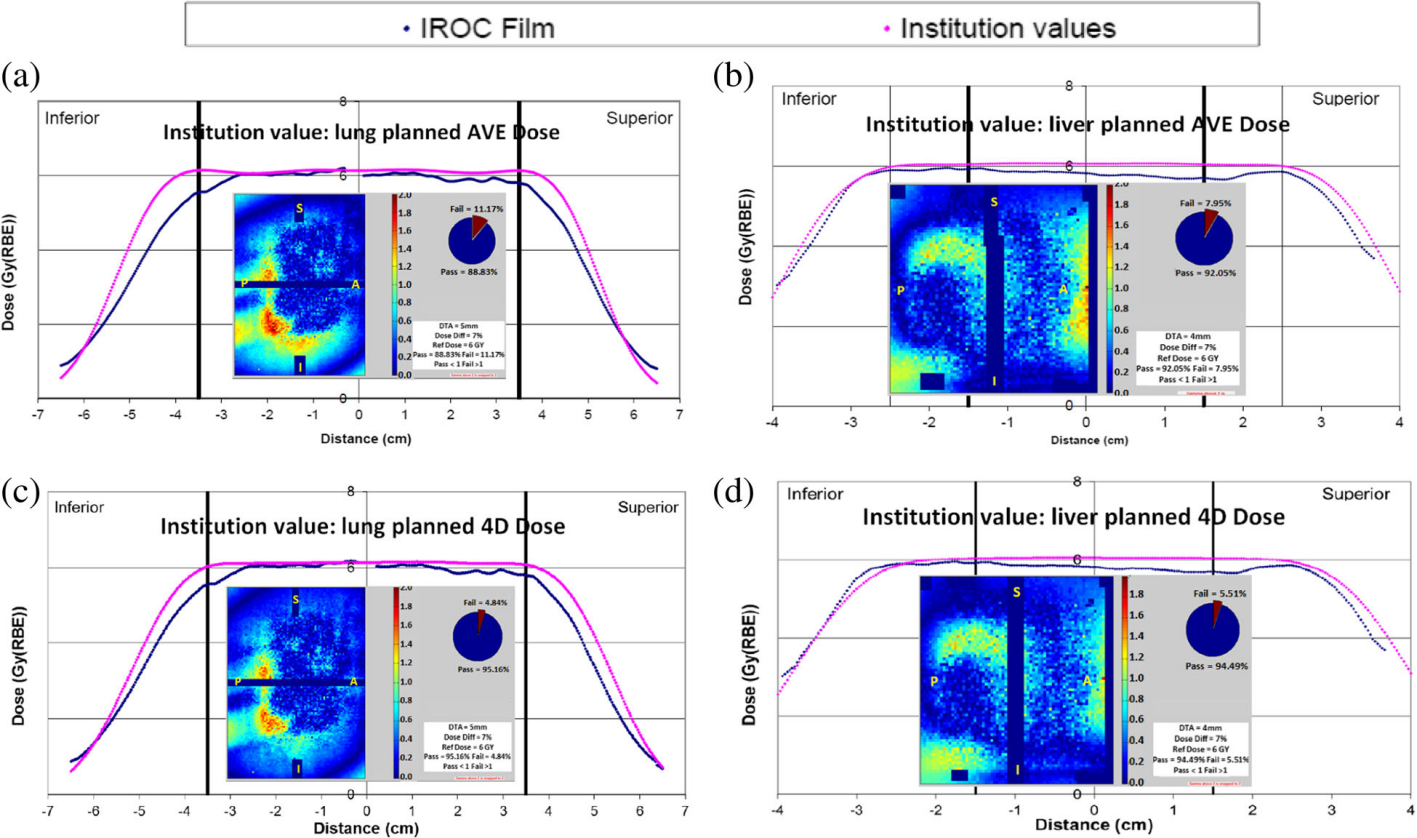
Planned AVE/4D dose compared with IROC-measured film dose in the center of the target in the target motion direction for IROC (**a**)/(**c**) lung, and (**b**)/(**d**) liver phantoms, respectively. Inserts are the 2D-2D gamma analysis results in the sagittal planes. Abbreviations: IROC, the Imaging and Radiation Oncology Core; AVE, average intensity; 4D, 4-dimensional; PTV, planning target volume; GTV, gross tumor volume

Figure 3 and Table 3 show IROC-reported film measurement analysis results for the treated lung phantom P2 and liver phantom P5. Both planned AVE and 4D dose passed IROC film dose comparison criteria. However, the IROC film 2D-2D Gamma index (lung:7% 5 mm, liver: 7% 4 mm) improved from 92 to 96% on average for the lung phantom, and from 92 to 94% for the liver phantom in the sagittal plane with a same high pass rate of 99% in the coronal plane using planned 4D dose compared with using planned AVE dose. The planned 4D dose matched the film-measured dose better in penumbra area in target motion direction compared with that of the planned AVE dose. With a stricter Gamma criterion of 3% 3 mm, the corresponding Gamma analysis pass rates improvements were 8% on average for the lung phantom and 3%/5% for the liver phantom in the sagittal/coronal plane (Table 3).

**Table 3:** Gamma pass rate comparing IROC-measured film dose with institution reported planned AVE and 4D doses for lung and liver phantoms

Phantom	Film plane	Gamma Index Lung 7% 5 mm Liver 7% 4 mm			Gamma Index 3% 3 mm	
		Criteria	AVE	4D	AVE	4D
Lung	Axial	≥80%	90%	95%	61%	64%
	Coronal	≥80%	96%	99%	70%	78%
	Sagittal	≥80%	89%	95%	65%	76%
	Average over 3 plane	≥85%	92%	96%	65%	73%
Liver	Coronal	≥85%	99%	99%	68%	73%
	Sagittal	≥85%	92%	94%	63%	66%

*Abbreviations*: *IROC* the Imaging and Radiation Oncology Core, *AVE* average intensity, *4D* 4-dimensional

### Simulated delivered dose compared with planned 4D and AVE doses

**Fig. 4:**
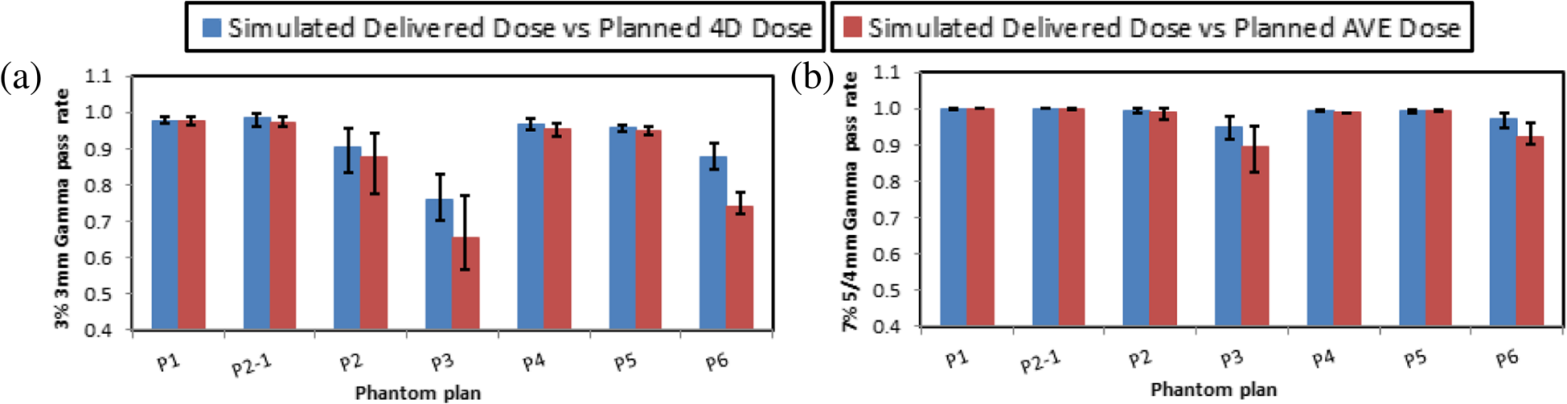
3D-3D (**a**) 3% 3 mm, and (**b**)7% 5 mm (lung) and 7% 4 mm (liver) Gamma pass rates for simulated delivered dose compared with planned 4D/AVE dose for the lung (P1, P2, and P3) and liver (P4, P5, and P6) phantoms. All doses are the sum of three 2 Gy(RBE) beam dose except P2–1 is the sum of three 1 Gy(RBE) beam dose twice. Values and error bars are the averages and ranges using 10 random beam starting phases for each beam. Abbreviations: 3D, 3-dimensional; 4D, 4-dimensional; AVE, average intensity

Figure 4 shows that the planned 4D dose matched the simulated delivered dose significantly better than the planned AVE dose for medium and large motion plans when 3% 3 mm 3D-3D Gamma pass rates were analyzed (*p ≤* 0.04), and for large motion plans when 7% 5/4 mm Gamma pass rates were analyzed (*p ≤* 0.01). The lower Gamma pass rate for the planned AVE dose when comparing with the planned 4D dose was due to the simulated delivered dose matched better in the penumbra regions for the planned 4D dose. Other cases did not show significant difference (*p* > 0.05) between using AVE dose and 4D dose as planned dose when the penumbra difference was much smaller than Gamma analysis criteria.

The dose difference between the simulated delivered dose and planned 4D dose caused by motion interplay varied largely depending on the beam starting phase (error bars in Fig. 4). For the lung phantom, Gamma pass-rate improved significantly when each beam was delivered twice compare to that of delivered once because the volume repainting mitigated the interplay effects. The *p* values comparing delivering each beam once with delivering each beam twice using ten random starting phases were 0.009/0.035 and 0.002/0.001 for 7% 5 mm and 3% 3 mm 3D-3D Gamma analysis comparing simulated delivered dose with planned 4D/AVE dose, respectively.

## Discussions

In this study, we evaluated the impact of using the planned AVE and 4D dose as reported planned dose to IROC to compare with film dose on the Gamma analysis pass rates for the proton mobile lung and liver phantoms. The IROC phantom tests are the end-to-end tests to verify the correctness and accuracy of the dose delivery to the phantoms in preparing safe patient treatment. Improving phantom dose evaluation accuracy can potentially help to improve patient dose evaluation accuracy because the phantom plan and treatment followed the same plan and treatment procedure as that of the patient. This study showed that IROC-measured film dose and simulated delivered dose agreed better with planned 4D dose than planned AVE dose, particularly in the dose penumbra regions. Therefore, report planned 4D dose to IROC for moving phantoms for IROC film analysis can improve dose comparison accuracy compared to report planned AVE dose.

When the moving phantom is treated using PBS beams, the delivered dose may vary due to target motion interplay effects. Therefore, evaluating and mitigating the motion interplay effect for PBS delivery is also necessary to pass Gamma-analysis criteria. In this study, volume repainting by delivering each beam two times (i.e., 6 times target volume repainting) to the lung phantom target showed improved simulated delivered dose 3D-3D Gamma analysis pass rate compared to that of delivered once (i.e., 3 times target volume repainting) (*p* < 0.035) and the phantom irradiated by delivering each beam twice passed IROC credential test, therefore, it was effective in mitigating interplay effect.

Due to the sharp motion interface between the chest wall and the dosimetry insert of the lung phantom, commercial deformable image registration tools did not achieve reasonable image registration result. Because the dosimetry inserts of the IROC phantoms were rigid and the film doses needed for the analysis was limited inside the dosimetry inserts, rigid registration was used to accumulate 4D dose in this study to avoid deformable registration uncertainty.

The scanned 4DCT image set measured a 1.3 cm motion for the lung phantom treatment planning due to the Anzai belt used for 4DCT acquisition suppressed the target motion magnitude, whereas the actual motion for the lung phantom treatment was 2.0 cm. During the 4DCT acquisition, the Anzai belt had to be tightened so that the pressure sensor in the belt could trace respiratory motion for 4DCT image sorting. In our practice, Anzai belt was used only for 4DCT, but not for motion management during patient treatment. Hence, to comply with our patient treatment procedure as recommended by IROC, Anzai belt was not used in the treatment delivery for IROC lung phantom. To ensure target coverage with actual treatment motion, the plan was optimized and evaluated for target coverage so that the plan is robust when the true motion (2 cm) was applied. Because 1.3 cm motion was used for treatment planning while the phantom had 2 cm motion for beam delivery, IROC film measured dose showed wider penumbras than the planned 4D dose in the target motion direction. The 4D dose penumbras were 13.1, 14.2, and 19.9 mm for 0.7, 1.3 and 2.6 cm lung phantom motion magnitudes, respectively (Table 2). The 4D dose penumbra increased as motion magnitude increased, and the linear fitting (R^2^ = 0.974) resulted in a penumbra of 17.5 mm for 2 cm motion, which is very close to the film measured penumbra of 18 mm in Fig. 3. Therefore, if the 2.0 cm motion were used for the lung phantom treatment planning, the penumbra of the planned 4D dose profile in the target motion direction would agree with the film measured dose better.

In this study, the center of the target alignment was used to compare the IROC film dose and simulated delivered dose with planned dose distributions. Because the films embedded in the phantoms moved with the targets, the center of the target alignment between the film dose and the planned 4D dose can be implemented using hardware alignment (i.e. the pin pricks on the films and targets) independent of which respiratory phase is used as the reference phase. However, hardware alignment doesn’t work when AVE dose is used as planned dose because the AVE dose is a static dose calculated on a synthetic CT image set and the pin pricks had blurred traces on AVE CT instead of discrete locations. When AVE dose was used as planned dose, IROC film analysis aligned the center of the IGTV on the AVE CT to the center of the GTV on the films located using pin prick positions on the films. Therefore, using 4D dose on a phase CT as planned dose for IROC motion phantoms has less dose alignment uncertainty for film dose analysis compared with using AVE dose as planned dose.

Previous studies showed the quenching effect of the Gaf-chromic film in the distal fall off of the proton beams [21–23]. In this study, the dose and Gamma analysis difference between the planned AVE and 4D doses were mainly in the lateral dose penumbra regions of the proton beams. Moreover, each PBS plan included multiple beams incident from different directions, which can mitigate the quenching effect. Finally, IROC has also carefully studied the film dosimetry when design the phantoms and limited the quenching effect to be within Gamma pass criteria with the design of the orientation of the film in the phantom (private communication). Therefore, we think the quenching effect should not affect our results.

Although this study was for PBS plans, our preliminary study showed that using planned 4D dose can improve IROC film dose Gamma analysis pass rate for the uniform scanning and double scattering plans as well compared to that of using planned AVE dose. Quantitative results in this study may not be directly applicable to those from other centers due to different machine characteristics [7, 8]. However, other centers may use the methodology described here in reporting planned dose to IROC to improve their dosimetric accuracy and improve phantom credentialing pass rates.

## Conclusions

Planned 4D dose is recommended to be used when institutions report planned dose to IROC for comparison with measured film dose for mobile tumors to improve Gamma analysis pass rate for IROC credentialing process.

## Data Availability

The datasets used and/or analyzed during the current study are available from the corresponding author on reasonable request.

## Abbreviations

2D: 2-dimensional
3D: 3-dimentional
4D: 4-dimensional
AVE: Average intensity
CT: Computed tomography
DVH: Dose volume histogram
GTV: Gross target volume
IGTV: Internal gross target volume
IROC: Imaging and radiation oncology core
Min-IP: Minimum intensity projection
MIP: Maximum intensity projection
PBS: Pencil beam scanning
PTV: Planning target volume
RBE: Relative biological effectiveness
T50: The 50% phase of a respiratory cycle
TPS: Treatment planning system
